# Synchronous Breast and Kidney Carcinomas Following Treatment for Hodgkin’s Lymphoma in Young Adulthood: A Case Report and Literature Review

**DOI:** 10.3390/jcm14248742

**Published:** 2025-12-10

**Authors:** Milan Žegarac, Milan Kocić, Dobrica Stević, Anđelija Cvetković, Ognjen Živković, Anđela Milićević, Marko Buta, Ivan Marković, Igor Đurišić, Zoran Kozomara, Zorka Inić

**Affiliations:** 1 Clinic of Surgical Oncology, Institute of Oncology and Radiology of Serbia, Pasterova 14, 11000 Belgrade, Serbia; kocic011@gmail.com (M.K.); d.stevic93@live.com (D.S.); andjelija.andjeli@gmail.com (A.C.); markobuta@gmail.com (M.B.); ivanmarkovic66@yahoo.com (I.M.); drigordjurisic@gmail.com (I.Đ.); kozomaraz@gmail.com (Z.K.); 2 Faculty of Medicine, University of Belgrade, Dr Subotića 8, 11000 Belgrade, Serbia; 3 Department of Pathology, Institute of Oncology and Radiology of Serbia, Pasterova 14, 11000 Belgrade, Serbia; drognjenzivkovic@yahoo.com (O.Ž.); andjela1204@hotmail.com (A.M.)

**Keywords:** multiple primary malignancies, Hodgkin’s lymphoma, breast cancer, renal cell carcinoma

## Abstract

**Background/Objectives:** Multiple primary malignancies (MPMs) are defined as the occurrence of two or more independent primary tumors in the same patient, histologically distinct and not of metastatic origin. Patients treated for Hodgkin’s lymphoma (HL) carry an increased risk of developing secondary malignancies, especially after chemotherapy and radiotherapy. The synchronous occurrence of breast and kidney carcinoma in this population is extremely rare. **Methods:** We present a 41-year-old female patient with a history of HL treated at the age of 23 with ABVD chemotherapy and supradiaphragmatic radiotherapy. **Results:** During staging for a newly diagnosed breast tumor (ER+/PR+/HER2+, pT1cN0), an incidental renal mass was identified and histologically confirmed as clear cell renal cell carcinoma (pT1aNxMx, G2). A multidisciplinary team performed simultaneous partial breast resection with sentinel lymph node biopsy and nephrectomy. The postoperative course was uneventful, and adjuvant systemic therapy was initiated according to oncological guidelines. **Conclusions:** Synchronous malignancies in HL survivors pose a clinical challenge, as they must be distinguished from metastatic disease and require coordinated therapeutic planning. Risk factors include prior radiotherapy, chemotherapy, genetic predisposition, and family history. This case highlights the importance of long-term surveillance of HL survivors, particularly young women, due to their elevated risk of secondary malignancies. Synchronous breast and kidney carcinomas after HL therapy are extremely rare and demand an integrated multidisciplinary approach. Early recognition and coordinated therapy are crucial for optimizing outcomes and contributing to a better understanding of the etiology and pathogenesis of multiple primary malignancies.

## 1. Introduction

The phenomenon of multiple primary malignancies (MPMs) was first described by Billroth in 1889, and the diagnostic criteria established by Warren and Gates in 1932 are still used in clinical practice today [1]. MPMs represent the occurrence of two or more independent primary tumors in the same patient, each with distinct histological features and not of metastatic origin. Based on the timing of their occurrence, MPMs can be classified as synchronous when the second malignancy occurs within six months, and metachronous when the interval is longer than six months [1]. The reported incidence of MPM ranges from 2.4% to 8%, increasing up to 17% over a 20-year follow-up period, with a rising trend in recent decades attributed to longer survival, advances in diagnostics, and modern therapeutic modalities [2].

Hodgkin lymphoma (HL) primarily affects adolescents and young adults (AYA, 15–39 years), with an age-adjusted incidence of ~3.4 per 100,000 person-years. Patients in this group generally have better 2- and 5-year relative survival rates, highlighting the need for long-term surveillance for late treatment-related complications, including secondary malignancies [3]. This elevated risk is mainly attributed to late toxic effects of treatment, including radiation therapy and chemotherapy, while genetic predisposition and impaired immune surveillance contribute further. Women who receive chest or mediastinal irradiation are particularly susceptible to secondary breast cancer, while chemotherapy increases the risk for leukemia, lung, gastrointestinal, bladder cancers, as well as soft tissue and bone sarcomas. Secondary malignancies are one of the most relevant causes of mortality in this population [4,5,6,7,8,9].

Breast cancer is the most common malignancy in women, accounting for 23.8% of all new cases, whereas kidney cancer is much rarer, comprising about 2.3% of new cases in women [10]. Although both tumors are relatively common individually, their synchronous occurrence in patients who have survived HL is scarcely documented. In patients without a history of HL, synchronous primary breast and kidney carcinomas occur sporadically and are mainly reported as individual case reports [11,12].

We present the case of a 41-year-old patient with a history of HL who subsequently developed synchronous breast and kidney carcinomas. This case contributes to a better understanding of etiology, diagnostic challenges, and therapeutic decisions in patients with MPM, and in young patients, it may indicate genetic predisposition or consequences of prior treatment.

## 2. Case Report

A 41-year-old premenopausal female patient, smoker, non-alcoholic, with ECOG performance status 0, and a positive family history (two maternal grandmothers and one paternal sister had breast cancer), presented with a palpable lesion in the right breast. Her history was significant for treatment of Hodgkin lymphoma, nodular sclerosis type, diagnosed in October 2006 at the age of 23. Given the limited disease at clinical stage IA, therapy was initiated according to the ABVD protocol: Adriablastin (doxorubicin) 40 mg, Bleomycin 15 mg, Vinblastine 10 mg, and Dacarbazine 600 mg per cycle. After four cycles of chemotherapy, she underwent supradiaphragmatic radiotherapy using the mantle field technique, with a total dose of 30 Gy delivered in 16 fractions, completed in June 2007. She continued with regular follow-up visits with her hematologist. During follow-up, she developed hypothyroidism, depression, and had an episode of acute pancreatitis.

**Figure 1:**
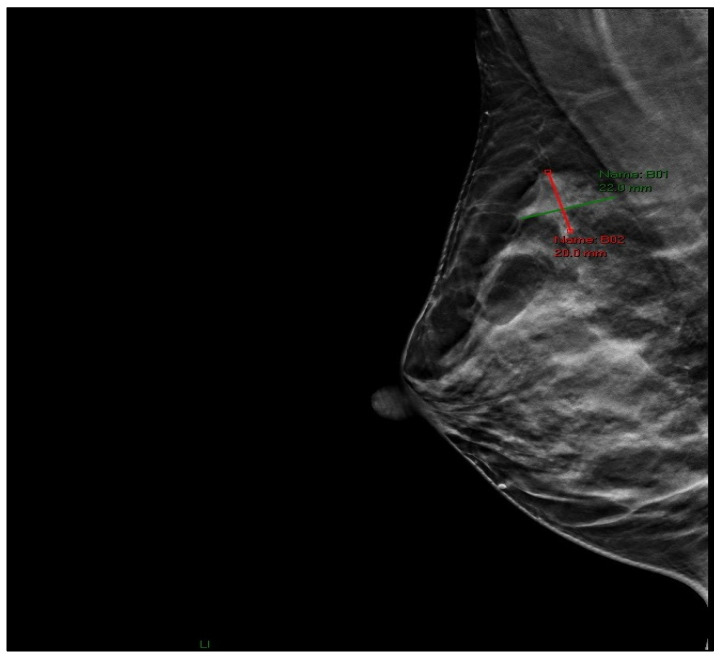
Mammographic image (RMLO projection) showing an area of architectural distortion in the upper quadrants of the right breast.

Physical examination revealed a palpable lesion in the right breast, cranially between 11 and 12 o’clock. Breast ultrasound showed a hypoechoic mass measuring 13 × 10 × 9.5 mm with irregular lobulated contours, a hypoechoic transition zone, and perifocal edema, without pathological axillary lymph nodes (BI-RADS 4). In June 2025, core needle biopsy confirmed invasive ductal carcinoma of the breast (HG 2, NG 2, ER 60%, PR 50%, HER2 3+, Ki-67 18%). Mammography confirmed a region of architectural distortion measuring 22 × 20 mm in the upper quadrants, prepectoral, 6 cm from the nipple, corresponding to the histologically verified tumor (BI-RADS 6) (Figure 1). The tumor was clinically staged as T1cN0M0.

**Figure 2:**
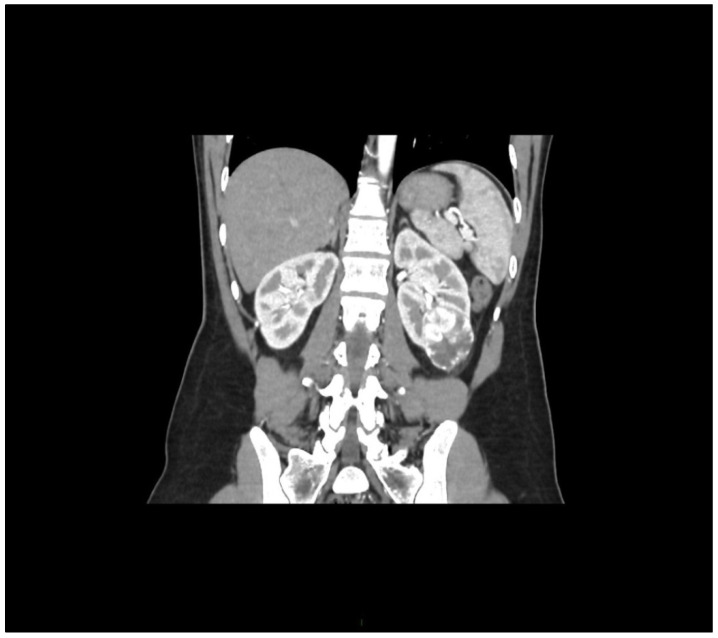
Arterial-phase multislice CT in the coronal plane shows a well-vascularized tumor at the lower pole of the left kidney.

During staging for breast cancer, an incidentally detected well-vascularized mass in the lower pole of the left kidney measuring 43 × 32 mm was identified on abdominal ultrasound. MSCT urography described a relatively well-defined soft tissue mass with intratumoral necrosis, measuring 45 × 40 × 39 mm, posterolateral to Gerota’s fascia. Radiologically, the findings were suggestive of renal cell carcinoma (RCC), although oncocytoma could not be excluded (Figure 2).

**Figure 3:**
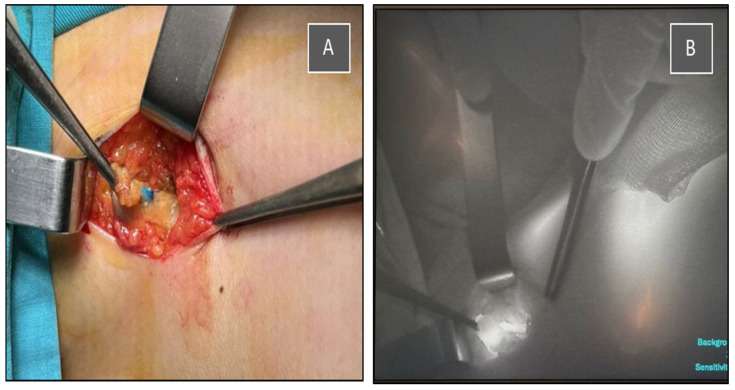
Intraoperative identification of the sentinel lymph node using a dual tracer method. (**A**) Blue-stained lymph node visualized after methylene blue injection. (**B**) Indocyanine green (ICG) fluorescence imaging of the same sentinel node using a near-infrared camera system.

**Figure 4:**
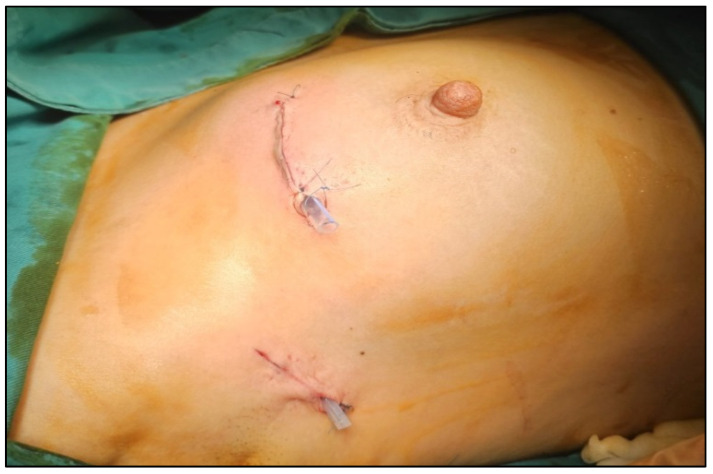
Intraoperative view showing both breast and axillary incisions after breast-conserving surgery and sentinel lymph node biopsy, with neat intradermal closure and good cosmetic alignment.

**Figure 5:**
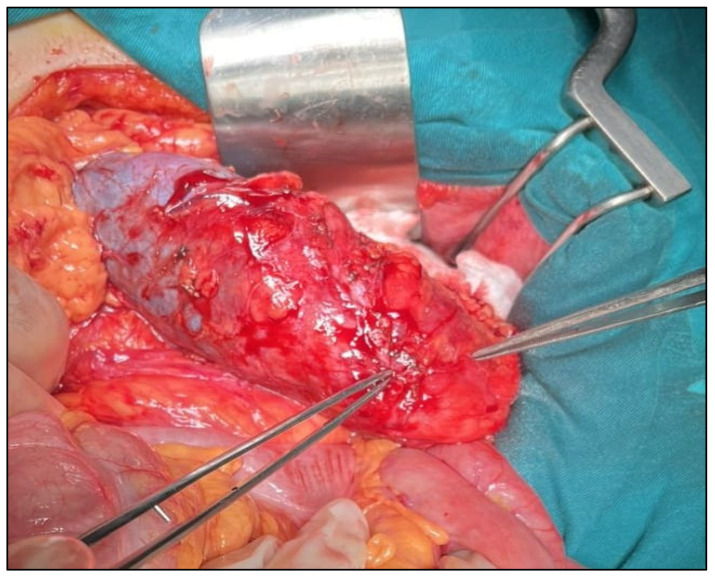
Intraoperative view of a tumor deforming the lower pole of the kidney.

The multidisciplinary team decided to perform, in the same procedure, a partial resection of the right breast with tumor localization and sentinel lymph node biopsy using dual tracers (indocyanine green—ICG and methylene blue), as well as left nephrectomy, which was successfully carried out in our institution (Figure 3, Figure 4 and Figure 5).

**Figure 6:**
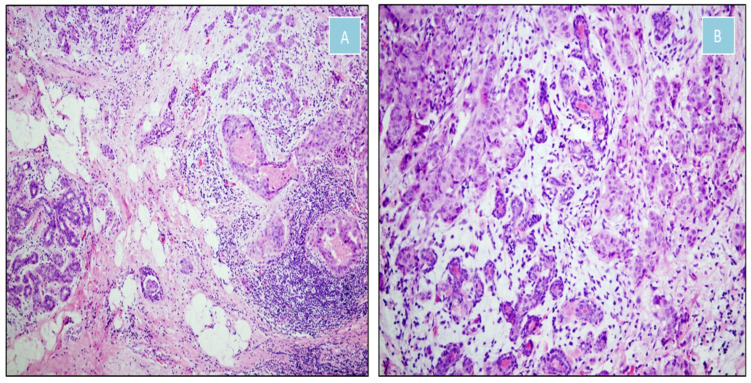
Moderately differentiated invasive ductal carcinoma of the breast (H&E). (**A**) Transition between malignant and non-neoplastic tissue (×100). (**B**) Malignant epithelial nests infiltrating fibrous stroma and intermingling with normal mammary ducts (×200).

Histopathology of the breast confirmed hormone-positive/HER2-positive invasive ductal carcinoma (GII, NG II) with a low proliferative index (Ki-67 15%), measuring 11 × 9 × 7 mm, with negative resection margins and negative sentinel lymph node. No lymphovascular invasion was observed. DCIS was present in approximately 5% of the tumor, with central necrosis, and no microcalcifications were detected (pT1cN0) (Figure 6).

**Figure 7:**
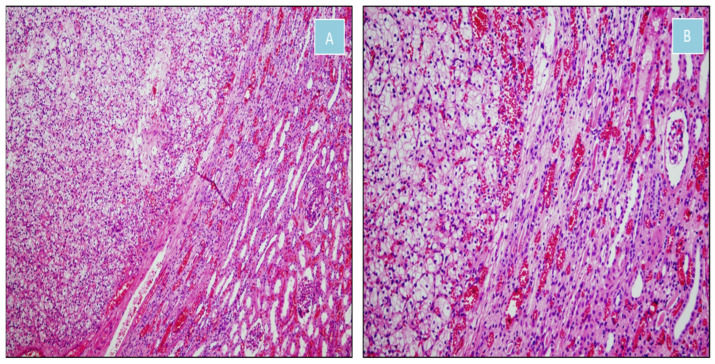
Clear cell renal cell carcinoma (H&E). (**A**) Nests of clear cells separated by a delicate vascular network, sharply demarcated from adjacent renal parenchyma (×100). (**B**) Transition between clear cell renal cell carcinoma and normal renal tissue (×200).

In July 2025, histopathology of the kidney revealed clear cell renal cell carcinoma (ccRCC), G2, measuring 25 × 25 × 15 mm, intrarenally localized, with central necrosis comprising ~15% of the tumor, without sarcomatoid or rhabdoid features, multifocality, or lymphovascular invasion; ureteral resection margins were free of malignancy (pT1aNxMx) (Figure 7).

The postoperative course was uneventful. The multidisciplinary team decided to continue treatment with adjuvant chemotherapy, consisting of 12 weekly paclitaxel cycles with the addition of LHRH analogs. After completion of chemotherapy, the patient will start adjuvant endocrine therapy with tamoxifen and receive postoperative radiotherapy to the remaining right breast. HER2-targeted therapy with trastuzumab is planned for 12 months. For kidney cancer, regular follow-up by a urologist is scheduled. Four months after surgery, the patient was asymptomatic, in good general condition, with preserved performance status, and adjuvant therapy had been successfully initiated.

## 3. Discussion

This case fulfills the criteria for multiple primary malignancies [1], as our patient was simultaneously diagnosed with invasive ductal breast carcinoma (ER+/PR+/HER2+, pT1cN0) and clear cell renal cell carcinoma (pT1aNxMx, G2) following treatment for HL at age 23 with ABVD and supradiaphragmatic radiotherapy. The observed increase in cases of multiple primary malignancies is largely attributed to longer patient survival and advances in diagnostics and therapy, as exemplified by this patient. Timely recognition of synchronous malignancies and their distinction from metastatic disease have direct implications for treatment planning and prognosis. Although such synchronous primary tumors are rare, the simultaneous development of breast and renal carcinoma in a single patient highlights the importance of long-term surveillance and awareness of multiple primary cancers.

The complexity of synchronous primary tumors, particularly in patients with a prior history of radiotherapy and chemotherapy, necessitates a multidisciplinary team (MDT) approach. Involvement of oncologists, surgeons, radiologists, pathologists, genetic counselors, and supportive-care specialists ensures comprehensive evaluation, accurate staging, and optimal individualized treatment planning. Coordinated MDT care reduces the risk of diagnostic and therapeutic errors, enables integration of multiple treatment modalities, and improves patient outcomes. Recent evidence supports that multidisciplinary care enhances survival, adherence to therapy, and quality of life in patients with complex cancer presentations [13]. Management of long-term therapy-related complications, such as hypothyroidism, depression, and pancreatitis, requires engagement not only of oncology specialists but also other relevant healthcare professionals, including endocrinologists, psychiatrists, and gastroenterologists, underscoring the need for a comprehensive multidisciplinary approach [8].

Beyond management considerations, understanding the long-term risk of secondary malignancies in HL survivors is essential. Large epidemiological cohort studies and meta-analyses have shown that patients with a history of HL have a significantly increased risk of developing secondary malignancies, including both hematologic and solid tumors. Among solid tumors, breast, lung, and gastrointestinal cancers are most common, whereas RCC is relatively rare [4,5,6,7,8,9]. Early studies, such as Van Leeuwen et al. (1994), demonstrated that cumulative risk of secondary malignancies in HL survivors increases over time and may exceed 20% after 20–25 years from initial treatment [4]. More recent data from Schaapveld et al. (2015) [5] confirm and extend these findings, reporting a standardized incidence ratio (SIR) for all secondary malignancies up to 4.6 compared to the general population, with cumulative risk reaching almost 50% over 40 years of follow-up. Collectively, these studies highlight that the long-term risk for any secondary malignancy remains high even after 35 years, with a SIR of 3.9. Notably, while the SIR for solid secondary tumors decreases with increasing age at diagnosis, breast cancer remains one of the most significant long-term risks in female HL survivors, with an absolute risk increase of ~54 cases per 10,000 person-years, representing approximately 40% of the total additional risk for any secondary tumor in women [5].

A large meta-analysis of 24,505 female HL survivors found a markedly increased risk of secondary breast cancer (SBC), with 957 cases identified over a median follow-up of 14.9 years and a pooled relative risk (RR) of 8.23 (95% CI, 5.43–12.47). The risk was highest in patients diagnosed with HL at ≤15 years (RR 68.7), while no significant increase was observed in women diagnosed at ≥40 years. The median latency to SBC was 17.7 years, with peak risk 15–19 years post-treatment. Radiotherapy was the dominant risk factor—RR 4.70 for radiotherapy alone and 5.65 for combined radiotherapy and chemotherapy—whereas chemotherapy alone did not significantly increase risk [6].

Understanding the long-term risk of secondary malignancies is essential, but it is equally important to consider the underlying etiologic factors that contribute to this risk, including treatment modalities, age at therapy, and biological characteristics of the tumors. The etiology of synchronous secondary malignancies is multifactorial and complex. Radiotherapy, particularly supradiaphragmatic (mantle field), applied at a younger age, is strongly associated with an increased risk of secondary cancers, especially breast cancer. Swerdlow et al. (2012) [7] reported an SIR of 5.0 for breast cancer in irradiated patients, indicating a fivefold increased risk compared to the general population. The highest risk was observed in women irradiated during adolescence; in patients irradiated at age 14, the SIR reached 47.2 and remained elevated for decades. The cumulative absolute risk for patients receiving ≥40 Gy mantle radiotherapy in youth can reach nearly 48% over 40 years of follow-up. Interestingly, the same authors showed that alkylating chemotherapy reduces the risk of secondary breast cancer, but only in women receiving radiotherapy after age 20, whereas the risk remains high in younger women [7]. These findings highlight that both age at exposure and treatment modality critically influence long-term risk, underscoring the importance of individualized surveillance strategies.

Chemotherapy exposure, particularly doxorubicin, has also been shown to independently increase the risk of secondary breast cancer in both adolescent and adult Hodgkin lymphoma survivors. In a large Dutch cohort, women treated with cumulative doxorubicin doses > 200 mg/m^2^ had a 1.5-fold higher risk, with risk increasing by 18% for each additional 100 mg/m^2^, emphasizing the need to consider chemotherapy exposure when planning long-term surveillance [9].

Young survivors of HL, particularly those treated with chest radiation before age 30, are at especially high risk of developing secondary breast cancer. This risk typically appears 8–10 years post-treatment and is higher in patients treated at younger ages, reaching levels comparable to those observed in BRCA1/2 mutation carriers, which underscores the need for early and intensive surveillance. Current guidelines recommend annual MRI and mammography starting at age 25 or eight years after radiation, whichever comes later. Breast cancers in this population are often estrogen receptor-positive and detected early, as was the case in our patient; however, overall survival remains poorer than in de novo cases, highlighting the importance of long-term monitoring and consideration of preventive strategies, including mastectomy or endocrine therapy [14].

A history of supradiaphragmatic radiotherapy before age 30, combined with an 18-year latency period since HL treatment, places our patient firmly within the high-risk group identified in the meta-analysis [6]. She received a cumulative doxorubicin dose of 160 mg, which is below the >200 mg/m^2^ threshold associated with significantly increased chemotherapy-related risk [9]. Nevertheless, the combination of early-age radiotherapy, prolonged latency, and even lower cumulative anthracycline exposure likely contributed to the development of SBC. These findings underscore the importance of long-term surveillance in HL survivors, including early and regular breast cancer screening with MRI and mammography, as recommended by the current guidelines [14].

Additionally, genetic predisposition, impaired immune surveillance, hormonal factors, and environmental exposures contribute to the risk of secondary malignancies [2]. A broader review of genetic predisposition in multiple primary malignancies, including BRCA mutations, Li-Fraumeni, and Cowden syndromes, is presented by Imyanitov (2023) and Lu et al. (2023), further confirming the role of inherited factors in the development of synchronous and secondary malignancies [15,16].

A positive family history of breast cancer in this patient prompted genetic testing, analyzing genes associated with hereditary breast and ovarian cancer (BRCA1, BRCA2, ATM, CDH1, CHEK2, PALB2, TP53, STK11, PTEN, NF1, RAD51C, and RAD51D) using next-generation sequencing (NGS). No pathogenic mutations (classes 4 and 5) were detected. This negative result indicates that the patient does not carry known high-risk hereditary mutations, but it does not exclude an increased risk due to family history, environmental exposures, or genetic factors not assessed by this test. In the context of prior chemotherapy and radiotherapy, her cancers may be related to late treatment effects rather than solely genetic predisposition. Continuous clinical follow-up of the patient and family members, including regular check-ups and preventive measures, remains indicated [17,18,19].

In our patient, management of SBC was guided by an MDT, including a medical oncologist, a radiation oncologist, and a surgical oncologist. She had previously received 30 Gy of mediastinal radiotherapy for HL, which required careful planning to safely perform breast-conserving surgery. Evidence from Bouziane et al. (2024) [20] supports that, even in patients with prior thoracic irradiation, breast-conserving surgery combined with appropriately tailored adjuvant therapy can achieve excellent local control and overall survival while minimizing long-term complications. This case underscores the importance of individualized, multidisciplinary strategies in optimizing both oncologic outcomes and quality of life for HL survivors [20].

Unlike breast cancer, which is a well-documented secondary malignancy after HL, the occurrence of renal cell carcinoma (RCC) in HL survivors is extremely rare. Epidemiological data on RCC following HL treatment are limited, with evidence mainly coming from isolated case reports [21], and RCC is not listed among the more frequent secondary malignancies in large HL survivor cohorts [4,5]. Therefore, the development of RCC in our patients likely represents a rare coincidental event rather than a predictable treatment-related complication.

While earlier reports suggested potential hormonal influences in renal tumorigenesis, contemporary evidence indicates that these are of limited relevance for clear cell RCC (ccRCC). Recent analyses, including the 2024 review by Ladurner et al., show that steroid hormone receptor expression in RCC is inconsistent and not clinically meaningful. The renal tumor in our patient is therefore more consistent with a sporadic ccRCC, and current literature provides no evidence that prior chest irradiation or hormonal factors significantly contribute to this subtype [22].

Clear cell RCC is the most common histological subtype of renal cancer, accounting for approximately 75–80% of cases. Approximately 37–61% of RCCs are incidentally detected during diagnostic procedures for other conditions, such as abdominal ultrasound or CT, and in our patient, the kidney tumor was similarly discovered without clinical symptoms during breast cancer staging, consistent with the literature [23].

Synchronous second primary malignancies in patients with RCC are relatively uncommon but clinically recognized. In a recent cohort of 1129 RCC patients, 6.2% were found to have a second primary malignancy, and among these, approximately 37% were diagnosed synchronously with RCC, indicating that, although rare, synchronous tumors do occur in contemporary clinical practice. Among the second primary malignancies observed, breast cancer accounted for 17.8% of all cases. However, of the 13 breast cancers reported, only one was diagnosed synchronously with RCC, highlighting that the simultaneous occurrence of breast carcinoma and RCC is rare but documented [24].

In our patients, the concurrent diagnosis of hormone receptor-positive breast cancer and RCC fits the definition of a synchronous second primary tumor. Although such constellations are infrequent, epidemiological data support that they are plausible and should be considered independent primary malignancies rather than metastatic lesions. Recognizing the possibility of synchronous tumors is essential for accurate diagnosis, staging, and management.

In a large retrospective cohort of 109,054 patients, 1.63% developed a second primary malignancy; among these, 29.1% were synchronous and 70.9% metachronous. Notably, metachronous tumors were associated with a better prognosis compared to synchronous tumors, consistent with other reports in the literature [25]. Although synchronous tumors are less common, the literature analysis shows that they represent a significant clinical challenge due to the need for coordinated diagnostics and simultaneous treatment planning. In patients with a history of Hodgkin lymphoma, synchronous malignancies are even rarer, further highlighting the scarcity and complexity of such cases. In our case, the multidisciplinary team enabled simultaneous surgical treatment of both tumors with an uneventful postoperative course. Timely diagnosis and coordination of therapy allow for integrated planning of surgical and adjuvant treatment, which is crucial for achieving optimal outcomes in these rare clinical situations.

## 4. Conclusions

Synchronous malignancies in HL survivors represent an extremely rare but clinically significant phenomenon. This case emphasizes the importance of a comprehensive, multidisciplinary approach in treating patients with a history of HL and concurrent primary tumors, as well as the need for long-term monitoring of high-risk patients for early detection of secondary malignancies. Early recognition and coordination of therapy are key to optimizing outcomes, while an individualized treatment plan is essential. This case contributes to the literature on rare combinations of synchronous tumors and underscores the necessity of careful follow-up of HL survivors, particularly young women and those with a positive family history, for timely detection of secondary malignancies. Studying synchronous malignancies can provide valuable insights not only for clinical evaluation and planning further treatment but also for understanding the etiology and pathogenesis of these tumors, as well as for developing future treatment strategies, including effective screening and surveillance protocols, aiming for optimal patient management.

## Data Availability

The original contributions presented in this study are included in the article. Further inquiries can be directed to the corresponding authors.
